# Eco-Efficient Recycling of Printed Circuit Boards

**DOI:** 10.3390/ma19112289

**Published:** 2026-05-28

**Authors:** Tomasz Suponik, Dawid Franke, Umut Kar, Paulina Gołuch, Maciej Mrówka, Maria Holuszko

**Affiliations:** 1 Faculty of Mining, Safety Engineering and Industrial Automation, Silesian University of Technology, Akademicka 2A, 44-100 Gliwice, Poland; tomasz.suponik@polsl.pl (T.S.);; 2 Department of Earth and Environmental Engineering, Columbia University, New York, NY 10027, USA; uk2182@columbia.edu; 3 Faculty of Materials Engineering and Digitalisation of Industry, Silesian University of Technology, Krasińskiego 8, 40-019 Katowice, Poland; maciej.mrowka@polsl.pl; 4 Norman B. Keevil Institute of Mining Engineering, The University of British Columbia, Vancouver, BC V6T 1Z4, Canada; maria.holuszko@ubc.ca

**Keywords:** circular economy, economic analysis, LCA, phytotoxicity, physical separation in recycling, polymer composites, printed circuit boards

## Abstract

This article presents a technology for the physical recycling of printed circuit boards (PCBs) that is consistent with the principles of circular economy and sustainable production. A life cycle assessment (LCA) was performed for PCB recycling using shredding, grinding, and physical and physicochemical processes such as electrostatic separation, gravity separation, and flotation for the separation of metals and plastics. Based on this assessment and the selectivity criterion, electrostatic separation was found to be the best separation method, followed by shredding and cryogenic grinding. For this option, the yield of metals and plastics was 25.1% and 72.5% of feed, respectively, while the yield of the middling’s product (mixture of metals and plastics) was only 2.4%. Furthermore, the financial benefits of recycling, including economics of the business case and the environmental benefits are presented. The possibility of using non-metallic fraction (plastic) generated during recycling as an additive in the production of composite materials was also assessed. The results suggest that low filler contents (2.5–5%) provide a compromise between maintaining mechanical performance and improving hardness and tribological properties. Physical recycling technology is a pretreatment method for WPCB, complementing conventional chemical recycling methods. The global warming potential for the entire physical and chemical process is then lowered by about 70%, due to the smaller mass of input material going to the downstream metallurgical processes.

## 1. Introduction

Waste electrical and electronic equipment (WEEE) is currently one of the fastest growing waste streams. According to the United Nations Institute of Training and Research (UNITAR), 62.3 Tg of WEEE was generated in 2022, while WEEE generated and reported in 2019 was 53.6 Tg, valued at USD 57 billion [1]. This waste stream is estimated to grow to 74.7 Tg in 2030 and 110 Tg in 2050 [2]. The rapidly growing volume of this waste is becoming a challenge for companies looking to recover valuable substances. A common element found in almost all WEEE are printed circuit boards (PCBs). The most used are FR-4 type PCBs, which are constructed from a substrate made of fiber glass and epoxy resin. The FR-4 waste printed circuit board (WPCB) contains embedded metallic layers containing large amounts of metals such as Cu, Fe, Al, Sn, rare earth elements, Ta, Ga, and others from the lanthanide group, and the precious metals Au, Ag, and Pt [3]. WPCB FR-4 also contains metals hazardous to human health and the natural environment, such as Cr, Pb, Be, Hg, Cd, Zn and Ni [4,5]. To protect the environment, WPCB should be processed according to the principles of circular economy and sustainable production. Concentrations of metals contained in PCBs are tens or even hundreds of times higher than in natural ores [6,7]. The average metal content in PCB is: Cu—20%, Al—2%, Pb—2%, Zn—1%, Ni—2%, Sn—4%, Ag—0.20%, Au—0.10%, Pd—0.005%, and Pt—0.0015% [4,8]. The most valuable metals in WPCBs are gold, palladium, silver, and copper, and the recovery of these metals can bring significant economic benefits for recycling [9]. The value of metals per ton of PCB is: Cu—USD 1770, Al—USD 51, Pb—USD 39, Zn—USD 30, Ni—USD 305, Sn—USD 1139, Ag—USD 1928, Au—USD 84,556, Pd—USD 1532, Pt—USD 454 [10]. Therefore, WPCB recycling is necessary for reasons of resource conservation and raw materials safety.

However, it should be noted that the environmental impact of inadequate WPCB recycling may be greater than that of extracting metals from ore deposits [11]. To protect the natural environment, WPCB should be recycled according to the principles of a closed-loop economy and sustainable production. These principles should be implemented in the design stage of recycling technology, meaning that they should be efficient, have a low environmental impact, and do not contribute to the depletion of natural resources. A technology that incorporates these criteria into account can be described as eco-efficient [12]. Implementing eco-efficiency principles in the development phase of WPCB recycling technologies can contribute, among other things, to lower material consumption, reduced waste and reduced emissions, and ultimately increase the competitiveness and improve an image of companies through sustainable solutions and environmental progress [13]. Therefore, the development of eco-efficient WPCB recycling technology represents huge potential for e-waste recyclers. It is also an opportunity to implement low carbon recycling technologies to reduce the environmental impact, as well as to be able to provide metals from secondary sources, thus reducing their extraction from natural resources.

Because of the complex structure of WPCBs, their recycling is a complex process that requires many steps to prepare adequate feedstock for metal extraction. The most important of these steps is the dismantling, which involves removing reusable components and/or decontaminating by removing toxic substances from the surface of the WPCB. Depending on the recycling method adopted, components that could interfere with further processes are also removed from the WPCB. A WPCB prepared in this way can become a suitable feedstock for the downstream recovery processes, which can be carried out either by chemical methods and/or a combination of both chemical and physical methods that are based solely on mechanical separation. The most common recycling methods for WPCBs are chemical metal extraction, such as hydrometallurgy [6] or thermal processes such as pyrometallurgy, including low temperature pyrolysis and plasma incineration. The common hydrometallurgical processing involves metal dissolution in acids, while pyrometallurgy uses high-temperature thermal processes to produce metals. In addition to the high complexity of these methods and their associated high level of emissions to the environment, there is also a loss of non-metallic parts that could be used for manufacturing of other products, which are otherwise lost into the residue and become waste after processing. The disadvantage of the hydrometallurgical method includes air and water pollution by hazardous substances, while the pyrometallurgical method involves the emission of greenhouse gases and flue gases into ambient air. Both methods generate hazardous waste. An alternative approach is processing of WPCBs starting with physical separation methods, including electrostatic separation, gravity separation, magnetic separation, and any combination of abovementioned methods to generate reduced volumes for a more intensive downstream processing by hydro- or pyrometallurgy [14]. These physical separation techniques have a much lower environmental impact, but because of the difficulty of releasing metals (liberation) from the WPCB composite materials, which requires sometimes several technological processes to be employed, they are less commonly used. When using physical separation methods, more than one product is generally produced, i.e., a mixture of metals with high financial value, plastics with a small amount of fiberglass, and an intermediate, middling type of material, i.e., particles containing plastics and metals. The key is to minimize the middling product while producing the cleanest possible fractions of metals and plastics.

The aim of this work was to implement eco-effective WPCB recycling technology that allows us to generate profits by selling precious metals while utilizing recycled plastics to produce other consumer goods. In particular, the focus was on separating metals from plastics from ground WPCBs while considering their economic and practical application in industry while leaving a minimal environmental footprint. To assess the environmental impacts of different recycling processes, LCA was applied. By applying LCA, it is possible to select the process with the least environmental impact and identify hotspots where environmental impacts are most significant, as well as to investigate ways to mitigate these impacts. A preliminary economic analysis was also conducted to obtain information on the costs of physical recycling. Furthermore, the monetary value of recovered metals calculated per 1 million inhabitants of southern Poland was presented, as well as the possibility of using recovered during recycling non-metallic powders to produce other consumer or industrial goods such as composites. The effect of powder addition on the functional properties, such as hardness, abrasion resistance, and tensile strength, of the composite was also investigated in this paper. Because consumer products are intended for use in the human environment, an additional phytotoxicity test of the non-metallic powder was conducted to assess its possible impact on the environment.

## 2. Materials and Methods

### 2.1. Materials

#### 2.1.1. Material for Recycling

The materials used for recycling were popular motherboards such as Intel, MSI, Asus, Nvidia, and Gigabyte. The boards were dismantled from desktop computers from 2007-09. All of the PCBs belonged to the FR-4 design, in which the laminate was composed of fiberglass and epoxy resin [15]. The goal was to analyze the selectivity of different separation methods for the same group of PCBs. Therefore, further complementary research is necessary to verify whether the WPCB processing technology is suitable for recycling and disposal of WPCBs from modern electronic products.

#### 2.1.2. Characteristics of Polymers and Fillers for the Production of Composite Materials

This assessment considered three types of polymer matrix—silicones, epoxy, and polyester resins. Silicones can be used for several applications, including adhesives and sealants, encapsulants, gels, protective coatings, thermal management materials, device packaging materials, and wafer-level coatings. They have a combination of properties that contribute to providing proven long-term reliability and performance: unmatched thermal stability with wide operating temperature range, flexibility, moisture resistance, UV and chemical resistance, adhesion to many common substrates used in electronics, low ionic impurity, and compatibility with common processing techniques [16]. In general, epoxy polymers have high specific strength and hardness, high chemical resistance, good processability, are resistant to weathering, and are relatively inexpensive [17]. Polyester resin serves as a widely used matrix material because of its unique properties, making it a cornerstone in various industrial applications. It is used in construction to produce durable, lightweight, and corrosion-resistant structural elements. It boasts impressive mechanical strength, making it suitable for load-bearing applications, and durability against external factors such as moisture, chemicals, and temperature variations makes it ideal for harsh environments. Polyester resin is cost-effective compared to other resins such as epoxy or vinyl ester [18]. Three materials were used as a matrix to prepare the composites, as follows.

- Mould Star™ 30 (Smooth-On, Inc. Macungie, PA, USA) is a polymer material in a form of hard silicone, whose properties are as follows: pot life—45 min, cure time—6 h, Shore hardness—30 A, tensile strength—2.89 MPa, Young’s modulus—0.66 MPa, elongation at break—339%, die b tear strength—15.41 kN/m, useful temperature (max)—232 °C, useful temperature (max)—−53.89 °C. They cure without shrinkage through polyaddition process, creating stable and durable material that is tear resistant.
- AROPOL M 105 TB (INEOS Composites; Grandview Heights, OH, USA) is a thixotropic, pre-accelerated, orthophthalic-based polyester resin with a moderate gel time whose properties are as follows: HDT—66 °C, density—1.1 kg/dm^3^, viscosity at 23 °C—180 mPas, gel time at 23 °C—40 min, peak exotherm at 23 °C—110 °C. As an initiator BUTANOX M-50 (INEOS Composites) properties are as follows: peroxide content—35%, amount of active oxygen—8.8–9.0%, SADT—60 °C. It was designed for curing unsaturated polyester resins, vinyl ester resins, polyester gel coats, varnishes. It is characterized by a very low water content and lack of polar compounds.
- LG 120 (Havel; Cieszyn, Poland) is a low-viscosity standard laminating epoxy resin with excellent adhesion and very good heat resistance, with properties as follows: density—1.17–1.22 g/cm^3^, viscosity—700–900 mPa/s. HG350 (GRM Systems; Olomouc, Czech Republic) is a fast hardener with short curing time, even in thin layers and with lower exotherm, with properties as follows: density—0.98 g/cm^3^, viscosity—100–120 mPa/s. Properties of the system are as follows: gel time—at 25 °C under 1 h, elevated temperature resistance—at 23 °C (5–7 days) up to 50 °C.

An addition to the matrix used was a filler in the form of recycled WPCB plastics. The powder particles were not larger than 1.2 mm and more than 40% of the grain masses were smaller than 0.125 mm. Plastic consisted mainly of fibrous and needle-shaped particles [19].

### 2.2. Methods

#### 2.2.1. WPCB Recycling Technology

**Figure 1:**
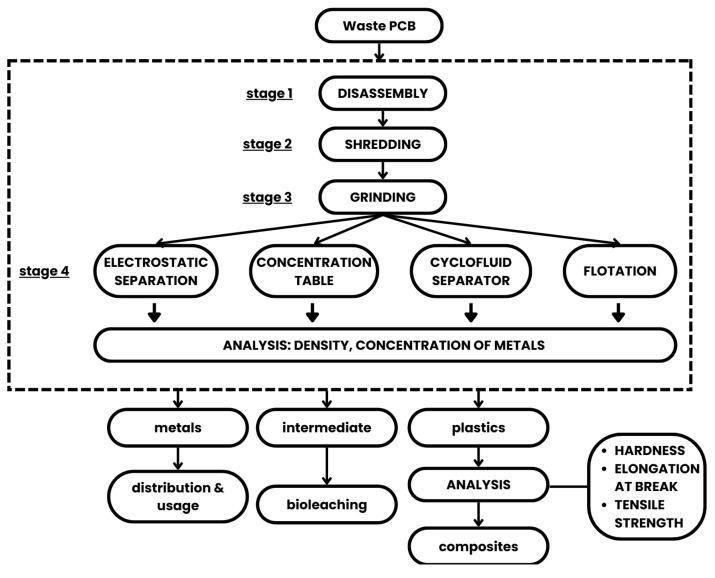
Flowchart of an eco-efficient WPCB recycling technology.

WPCB recycling technology using physical methods has been divided into 4 stages (Figure 1). The goal was to separate metals from plastics, while the first stage of the research was to remove components from the surface of the PCBs that may interfere with subsequent processes, i.e., sockets, resistors, transistors, processors, RAM disc, and others. Disassembly was carried out using handheld small tools [20]. A preliminary evaluation of components with high reuse potential was made; these were processors and their cooling systems, various types of connectors, and RAM memory. Dismantled WPCBs were crushed in a shredder to obtain pieces not larger than 1 × 1 cm [19]. Reducing the size of the WPCBs was necessary to prepare material for the fine grinding, aka milling. The proper release of metal particles from the rest of the WPCB composite matrix is one of the most critical factors for successful recycling physical methods to achieve efficient separation. In previous work by the authors [19], the use of liquid nitrogen was observed to have a positive effect on the WPCB grinding process. However, the cryocooling of the WPCB pieces could not take place directly in the mill’s working compartment, so this stage was divided into two parts. First, the cut WPCB pieces were cooled to below −150 °C using liquid nitrogen in a special liquid nitrogen tank. The frozen WPCB pieces were ground in a LMN-100 laboratory knife mill (Testchem LLC, Radlin, Poland) equipped with a 1 mm sieve. The result was generation of a WPCB powder with a maximum particle size of 1.5 mm. The grain size distribution was: 1.5–1.0 mm—0.4%, 1.0–0.71 mm—1.4%, 0.71–0.50 mm—10.2%, 0.50–0.36 mm—22.1%, 0.36–0.25 mm—14.2%, 0.25–0.18 mm—6.5%, 0.18–0.13 mm—5.3%, 0.13–0.09 mm—4.3%, <0.09 mm—35.6%.

Separation of metals from the WPCB composite was carried out using the following laboratory devices: (1) an electrostatic drum separator, (2) a shaking table, (3) a cyclofluid separator, and (4) a flotation machine. These devices were fed with the same material. The separation was optimized for all the devices in order to obtain the best possible separation results. A shaking table, cyclofluid separator, and flotation used water for separation, so the resulting products had to be dried.

The first separation process studied was electrostatic separation, which is based on differences in the surface charge storage capacity and conductivity of dry particles. In electrostatic drum separators, nonconductive particles adhere to a rapidly rotating drum, which is electrified by friction against a brush that also mechanically scrapes these particles (plastics). Conductive particles (metals) rapidly release their surface charge, allowing them to easily be “stripped” from the drum surface. To increase the efficiency of this process, the electrostatic separator was equipped with a high-voltage electrode that bombards the particles with electrons. A Boxmag Rapid Laboratory Electrostatic Drum Separator (Aston, Birmingham, UK) was used, which was designed to receive three products, i.e., metal, middlings, and plastics. Separation was carried out under the following conditions: drum rotating speed of 100 rpm, voltage of 17 kV, and distance between the electrode and the drum of 0.03 m [21].

The second method investigated was gravity separation, which was used to separate particles with different densities. It was decided to use two types of gravity separators that interacted with the particles in different ways. In both cases, the separation was performed in an aqueous medium. Separation was carried out using a laboratory shaking table equipped with a grooved surface. Separation was carried as follows: table load of 9 dm^3^/min (water suspension with material), water flow rate of the first nozzle of 5.7 dm^3^/min, water flow rate of the second nozzle of 5.4 dm^3^/min; table stroke of 1.5 mm, table movement frequency of 260 strokes/min, longitudinal inclination angle of 1° and transverse inclination angle of 6° [22]. The lightest particles (plastics) were stripped from the table surface as the fastest flowing, while the heaviest particles (metals) overcame the hydrostatic pressure of the liquid and were carried along the table surface resulting from its movement. Spray nozzles were placed on top of the table to increase the efficiency of the separation process.

The second device was a laboratory cyclofluid (elutriation) separator, in which separation was achieved by vertical fluidization of the particles, where the vertical movement of the liquid caused the particles to separate according to their density. The heaviest particles (metals) fell to the bottom of the separator, while the lightest particles were lifted to the surface. The principle of operation of the laboratory cyclofluid separator involved the use of a semi-industrial U-shaped cyclofluid separator with continuous movement [23]. This separator was equipped with a water-filled tank in which a cylinder was placed. The cylinder containing the WPCB powder suspension was closed at the bottom with a 0.5 mm-mesh sieve and performed a vertical reciprocating motion. This created a cyclical fluid thrust in the cylinder and allowed the particles to be separated by density. The following parameters were used: water volume of 13 dm^3^, cylinder stroke of 4 cm, and suspension movement frequency in the cylinder of 53 movements/min [22].

The final separation process studied here was flotation, which involved the separation of particles based on differences of hydrophobicity. The flotation process took place in aerated water so that hydrophobic particles could “stick” to air particles and float to the surface of the cell. Flotation was carried out using a laboratory flotation cell Mechanobr, from IMN Gliwice (Instrumentation Building Plant of the Institute of Non-Ferrous Metals, Gliwice, Poland), with a 1 L flotation tank. During the flotation process, a flotation reagent (dimethoxy dipropyleneglycol) was used at a concentration of 157 mg/dm^3^. The rotation speed of the rotator in the flotation cell was approximately 400 rpm, and the flotation time was 5 min [24].

After selecting the separation method, the final step was distribution and use. Plastics (waste) obtained as a result of WPCB recycling were used in the research as an additive in the production of composite materials, while the metal mixture (concentrate) can be sold to the smelters.

#### 2.2.2. Life Cycle Assessment (LCA) Methods

LCA study focused on assessing the environmental impact of different options for separating metals from plastics from WPCBs. LCA was carried out according to ISO 14040/43 standards [25,26,27,28], which includes four main phases; goal and scope definition, inventory analysis, impact assessment, and interpretation. The main objective of this LCA was to assess and compare the environmental impacts of four different separation technologies options for WPCBs. These options included electrostatic separation, flotation, and gravity separation on a shaking table and in a cyclofluid separator. The study aimed to identify the most environmentally friendly separation method that could be scaled up for industrial applications. The functional unit for this LCA was defined as the treatment of 1 kg of WPCB, which ensures consistent comparisons between different separation processes. In addition to the separation options, the environmental impact of the comminution processes was also evaluated to understand the impact of the overall process of recycling of WPCB from size reduction to separation stages.

##### System Boundary

**Figure 2:**
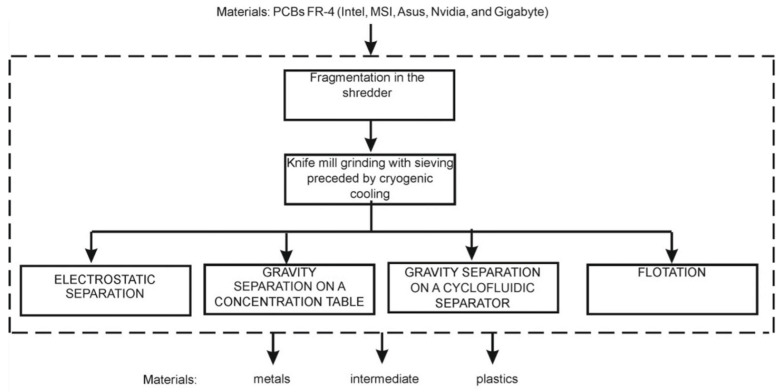
Flowchart of WPCB recycling for LCA, illustrating four methods for separating metals from plastics, including comminution steps.

The LCA system boundary covered the steps of the WPCB recycling process from fragmentation in the shredder to producing a saleable metal mixture (Figure 1). Each step is evaluated separately. As shown in (Figure 2), the WPCB recycling system included different scenarios with the steps as follows: fragmentation in the shredder, knife mill grinding with sieving preceded by cryogenic cooling and four separation options, including electrostatic separation, flotation, and gravity separation, on the shaking table and the separation in cyclofluidic separator.

The inventory analysis was carried out using the Ecoinvent 3.0 database, a comprehensive and widely recognized source of life cycle inventory data. The SimaPro 8 software was used to model processes and compile inventory data [29]. The inputs for each method were methodically recorded and analyzed. Data for shredding and cryogenic grinding, such as energy requirements and the usage of liquid nitrogen, were included along with the separation methods. The inventory Table A1 (Appendix A) is listed as the supporting information.

The environmental impacts of the recycling processes were evaluated using the Tool for the Reduction and Assessment of Chemical and Other Environmental Impacts (TRACI), developed by the US Environmental Protection Agency (EPA) [30]. TRACI is a midpoint-orientated technique that evaluates environmental burdens across various impact categories, making LCA results easier to interpret. Impact categories considered in this study were: ozone depletion (kg CFC-11 eq), global warming (kg CO_2_ eq), smog formation (kg O_3_ eq), acidification (kg SO_2_ eq), eutrophication (kg N eq), carcinogenic effects (CTUh), non-carcinogenic effects (CTUh), respiratory effects (kg PM2.5 eq), ecotoxicity (CTUe), and fossil fuel depletion (MJ surplus).

The results of the TRACI impact assessment were analyzed to identify the most environmentally sustainable option to separate metals from plastics and to demonstrate the overall environmental impact of the studied recycling technology. The findings were interpreted to provide information on the potential environmental benefits and shortcomings of each process.

#### 2.2.3. Using WPCB Recycled Plastic to Produce Composite Materials—Strength Parameters

In the case of plastics for the production of composite materials, the impact of the share of recycled powder in the matrix on the strength parameters of the final product was evaluated. The powder consisted mainly of fibrous and needle-shaped grains, which size ranged from less than 50 μm to over 1000 μm.

The composites were prepared by gravity casting with a filler (plastics obtained from recycling) content of 2.5, 5.0, 7.5 and 10.0 wt% and were marked for silicone, polyester resin, and epoxy resin as: 2.5_F, 5_F, 7.5_F and 10_F, respectively. Before fillers were introduced into the matrix, hardening agents and matrix cross-linking initiators were added. The silicones and resins with the above agents were then placed in a rotary mixer and the filler was gradually added while mixing at a speed of 150 rpm until the assumed filler contents was achieved. After the fillers were placed in the matrix, the resulting mixtures were placed in appropriate forms. For polyester resin, the molds were heated at approximately 80 °C for 24 h. Then 72 h after pouring, the samples were removed from the molds and subjected to mechanical and physical tests. Static tensile tests, Shore A-type hardness tests, and Schopper–Schlobach abrasion resistance tests were performed for samples made of silicone materials, while static tensile tests, Shore D-type hardness tests, and pin-on-disc tests were performed for resin materials. All tests were carried out at room temperature (22 °C) and 50% humidity. Tensile tests were carried out according to [31] for five samples (type 5-B) cut from the composites and silicone samples. The tests were carried out on an Instron 4465 tensile tester (Instron, Norwood, MA, USA) equipped with a mechanical contact extensometer. The test speed was 500 mm/min. The tensile strength and elongation at break were determined. Shore A and D hardness tests were carried out according to [32]. Measurements were made with a Shore A-type hardness durometer Zorn (Zorn Instruments GmbH & Co., Hansestadt, Germany). Five measurements were made in each sample. The abrasion test (pin-on-disc) for epoxy and polyester resign was performed on the CSM Tribometer Instruments (Needham, MA, USA) according to ASTM G99 [33]. The test samples were cylindrical in shape, 10 mm high and 12 mm in diameter. Before the test, the samples were cleaned with technical ethanol. The ball moving after the sample, with a dimension of 6 mm, was made of zirconium dioxide. The ball was pressed against the sample with a force of 20 N. The ball’s linear speed was 10 cm/s. The abrasion of the materials tested was defined as a change in the coefficient of friction (µ) on the 20 m road. For each group of materials tested, 5 measurements were made, for which the arithmetic mean was obtained. The abrasion wear of silicones was measured according to EN ISO 4649:2007 [34] on an APG Schopper–Schlobach apparatus (APG Germany GmbH, Friedberg, Germany). For the abrasion test, the arithmetic mean of three measurements was calculated.

#### 2.2.4. Using WPCB Recycled Plastic to Produce Composite Materials—Phytotoxicity Test

To determine the impact of recycled WPCB plastic on the surrounding environment, phytotoxicity tests were carried out on the leachate resulting from washing the powder with distilled water according to the PN-EN 12457-2:2006 standard [35]. The plastics were leached on a ROTAX 6.8 shaker for 24 h. The liquid/plastic ratio (L/S) in the tests was 10:1. The leaching test was carried out at 22 °C. The eulate was filtered prior to use.

Phytotoxicity tests were performed for two dicotyledonous plants: mustard (*Sinapis alba* L.) and cucumber (*Cuccumis sativus* L.) and one monocotyledonous plant: wheat (*Triticum* spp.). For comparison purposes, phytotoxicity was also determined for distilled water (control). For each species, the test was performed in petri dishes in 5 repetitions. For this purpose, 10 mL of sample (distilled water and leachate) was placed on each plate with filter paper and 10 seeds were seeded. After 5 days after sowing, the number of germinated seeds was counted and 14 days after sowing, the lengths of the stem and root were measured. The tests were carried out according to PN ISO 11269-2 [36] and PN-ISO 11269-1 [37].

## 3. Results

### 3.1. WPCB Recycling

The results of the separation of metals from plastics using an electrostatic drum separator, a shaking table, a cyclofluid separator and a flotation machine have already been discussed in previous papers [19,22,24]. The aim of this study was to select the most efficient and environmentally friendly method (see LCA) among those analyzed and to retest the best one using a different and larger amount of feed (15 kg).

**Figure 3:**
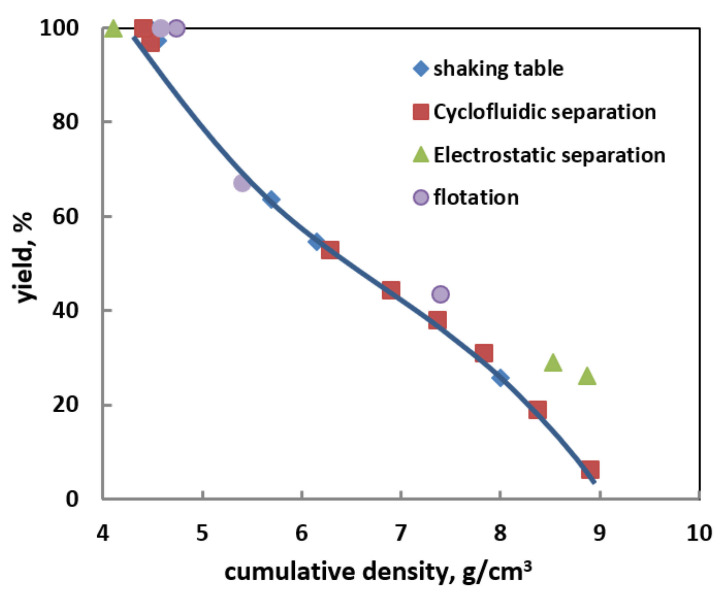
Correlation between product cumulative yield and cumulative density of separation products.

To demonstrate which of the methods is the most selective, it was proposed to use the specific density of the products as a parameter characterizing the quality of the obtained separation products and to plot the dependence of the product yield on the cumulative density of the separation product, i.e., the upgrading curve (Figure 3). The results indicate that electrostatic separation was the most selective method, and therefore only this method is shown in (Figure 1) and the separation was repeated for a larger amount of feed.

Three products were: concentrate, intermediate, and waste. Concentrate and waste yield were 25.1% and 72.5% of feed, respectively, with densities of 8.94 g/cm^3^ and 2.15 g/cm^3^. These confirm the high purity of the resulting metal concentrate and plastic as waste product. The intermediate or middling’s product, whose yield was only 2.4%, consisted mainly of mixed in nature particles that cannot be physically separated. The density of this product is 5.41 g/cm^3^. To release the metal from this fraction, these particles would need to be re-crushed under the appropriate conditions to liberate the metal fraction. Alternatively, to process these middling’s, another method such as bioleaching with the *Acidithiobacillus ferrooxidans* can be used, which is able to dissolve the metals contained in WPCB [38]. This method is usually used as an auxiliary method due to the mechanics of the process and its long kinetics [38,39,40]. However, for such a small amount of intermediate product, a small bioleaching installation would be an effective complement to complete the WPCB recycling process.

**Figure 4:**
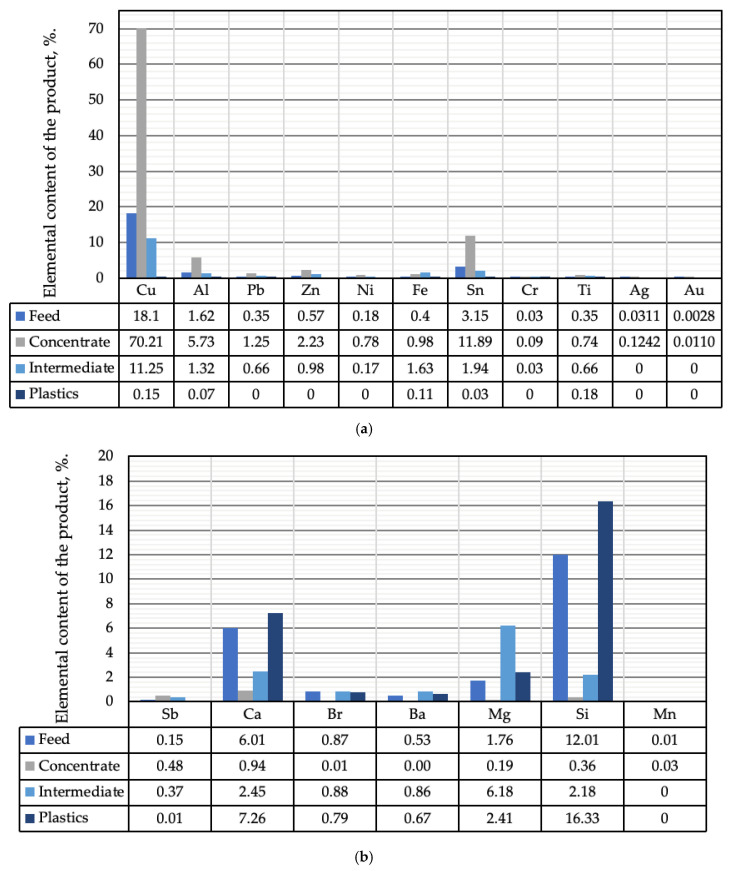
Chemical composition of feed and electrostatic separation products: (**a**) valuable elements, (**b**) non-valuable elements (value “0” i.e., below detection threshold).

The chemical analysis of electrostatic separation products indicates that more than 94% of valuable metals (that is, Cu, Al, Pb, Zn, Ni, Fe, Sn, Cr, Ti, Ag, Au) are found in the concentrate (Figure 4). It could be assumed that because of the multilayer structure of the PCB, it is advantageous to crush to obtain the smallest possible particles. Wu et al. 2008, however, report [41], that very fine particles, i.e., less than 0.125 mm, may contribute to inefficient electrostatic separation due to the particle aggregation effects on the drum and electrode surface. In the work of Wu et al. 2009 [42] it was shown that this effect can have a significant impact on the stability of the separation process itself. The settling of plastic dust on the electrode surface was observed in the study presented in their paper. However, the aggregation effect was observed to be strongly reduced when the material was previously ground using cryogenic temperatures during milling.

### 3.2. Life Cycle Assessment (LCA)

**Figure 5:**
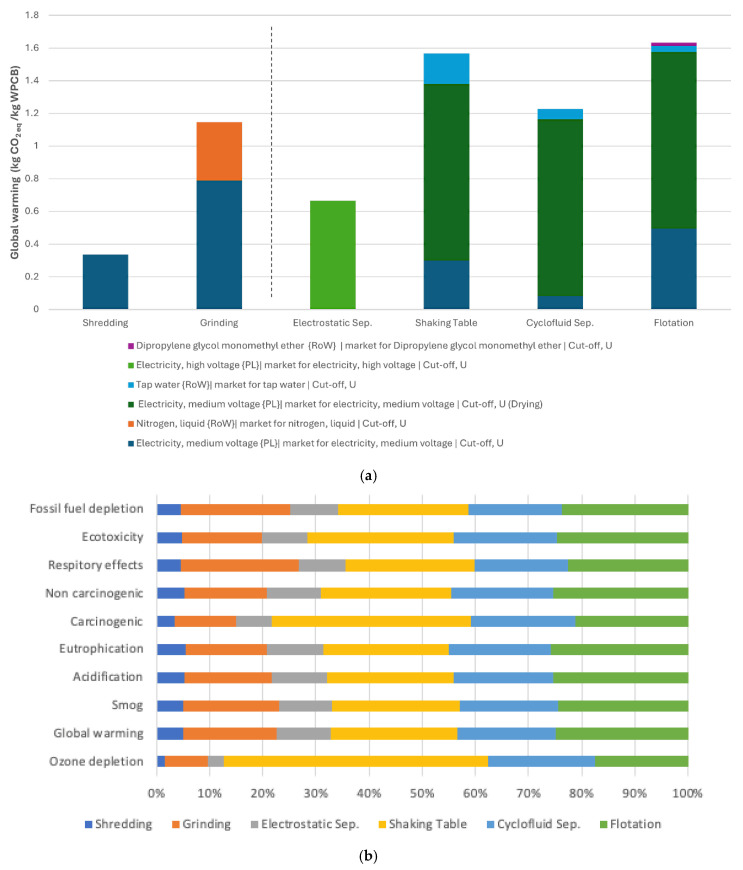
(**a**) Global warming potential of four WPCB separation options along with the comminution processes. (**b**) TRACI midpoint categories showing the relative contribution of all WPCB recycling processes.

The global warming potential (GWP) of the four WPCB separation options shows that they are ranked from most to least influential as follows: flotation, shaking table, cyclofluid separator, and electrostatic separation (Figure 5a). The latter has an impact of 0.665 kg CO_2eq_/kg of WPCB. The several times higher GWP for flotation, shaking table, cyclofluid separator, is mainly due to the need to dry the products obtained from these processes. For flotation, the impact of tap water consumption and flotation reagent consumption is very small, while the major GWP comes from energy used for mechanical flotation cell operation.

From the environmental impact point of view, it can be concluded that electrostatic separation was shown to be the best option for WPCB recycling. While the GWP for the entire physical recycling of WPCB, including the comminution processes, is 2.147 kg CO_2eq_/kg of WPCB. This is mainly due to energy consumption for the electricity and, to a lesser extent, the use of liquid nitrogen before grinding. Although its use does still not allow the extraction of metals in the form of pure and fully liberated particles, the resulting product is a mixture of metals from which individual metals can only be obtained by chemical processing. Physical recycling separates plastics that can be utilized further, since they make up 70% of the total mass of WPCB. As a result, approximately only 30% of the material would go to chemical extraction by leaching.

Currently, WPCB recycling methods are often based only on chemical leaching methods such as hydrometallurgy or by thermal processing by pyrometallurgy. Commonly, these methods do not involve pretreatment to remove plastics, resulting in significant greenhouse gas emissions and waste generation, as well as the consumption of energy and reagents [43]. For comparison, GPW for chemical recycling of WPCB by hydrometallurgical method using glycine solution as an environmentally friendly main leaching agent is 12.4 kg CO_2eq_/kg WPCB [44]. Assuming that in the case of physical recycling 70% less mass would go to hydrometallurgy, the GPW for it would be lower by 8.66 kg CO_2eq_/kg WPCB. In this way, the GPW for physical recycling is compensated for, and the plastics generated in it can be used to produce other consumer goods. As a result, hydrometallurgy recycling can also produce less waste and use fewer leaching reagents.

The remaining midpoint categories shown in Figure 5b follow trends similar to GPW, with flotation, shaking table, and cyclofluid separator accounting for approximately 70% of total impacts. The exception is ozone depletion, for which the shaking table is close to 50% burden.

### 3.3. Strength Parameters of Composite Materials

#### 3.3.1. Tensile Tests of Composite Materials

**Figure 6:**
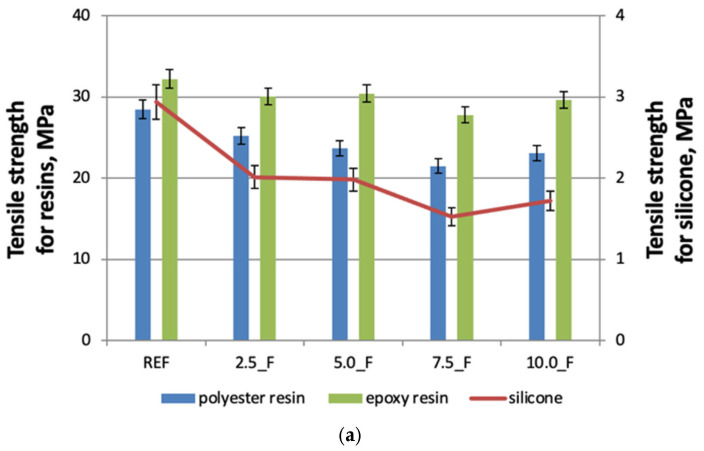

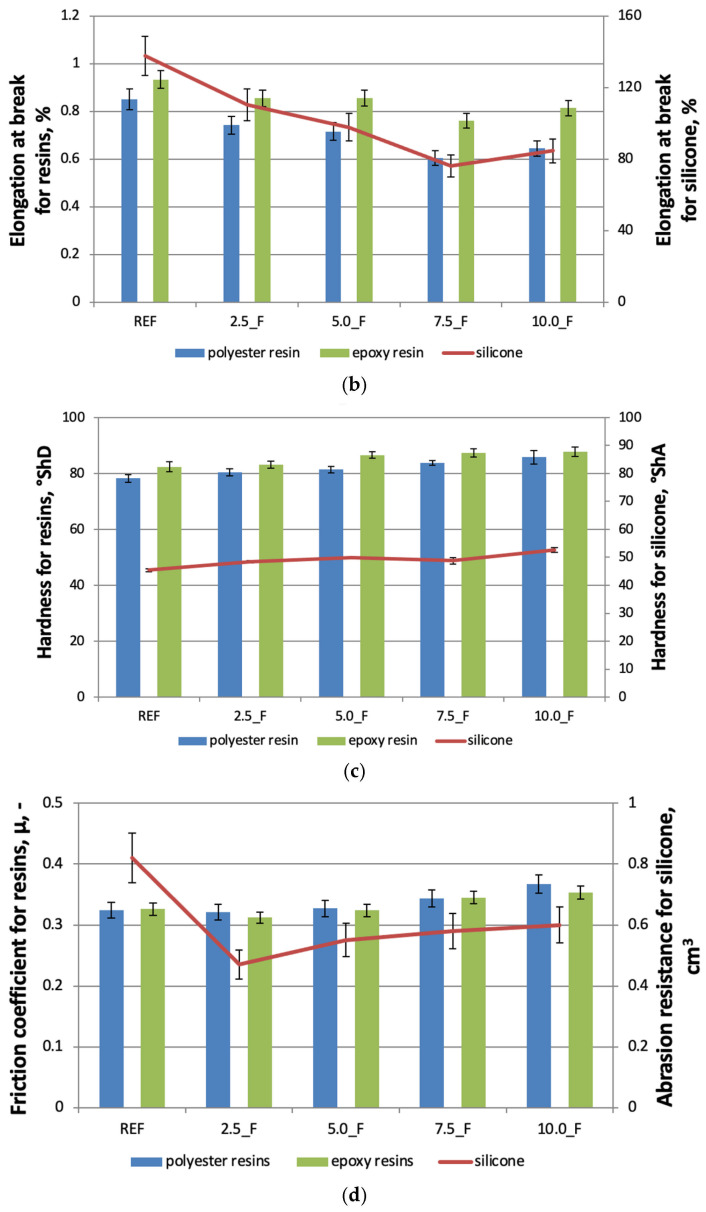
Test results of strength parameters of composite materials. (**a**) Tensile strength for epoxy and polyester resins as a matrix in MPa and for silicone as a matrix in MPa, (**b**) elongation at break for epoxy and polyester resins as matrix in % and for silicone as matrix in %, (**c**) hardness for epoxy and polyester resins as matrix in °ShD and for silicone as matrix in °ShA, (**d**) friction coefficient for epoxy and polyester resins as matrix and abrasion resistance for silicone as matrix in cm^3^.

The results of mechanical tests of composite materials obtained by adding plastic powder obtained from recycled WPCB to the matrix and for pure epoxy, polyester resins and silicone (REF) are presented for tensile strength in Figure 6a (for silicone and resins) and elongation at break in Figure 6b (for silicone and resins).

For silicone used as a matrix, the highest tensile strength (2.94 MPa) was found in a sample made of material without filler. In the case of silicone-based composite materials with the addition of PCB powder, the tensile strengths were lower than those of the reference samples (REF). Tensile strength values slowly decreased with increasing filler content in the composite. The lowest value was measured for samples with a PCB powder content of 7.5%. The tensile strength was 1.53 MPa and was lower than the reference value by 1.41 MPa. This value was 48% lower than for the sample with the highest tensile strength measured for the reference sample (2.94 MPa). Such a significant reduction in tensile strength, also observed in Figure 6a, indicates that the introduced filler does not act as a reinforcing phase but rather as a stress concentrator, which promotes crack initiation at the matrix–filler interface. This suggests limited interfacial adhesion and ineffective stress transfer, particularly in the case of silicone, where the elastomeric nature of the matrix leads to a higher sensitivity to structural discontinuities.

Tensile strength also decreased when PCB powder was added to polyester and epoxy composites. The lowest value was also obtained for the samples with the addition of powder in the amount of 7.5%. This value was 21.5 and 27.8 MPa for the polyester and epoxy resins, respectively, while for the reference samples it was 28.5 and 32.2 MPa. Therefore, the tensile strength values were lower than the reference value by only about 15% for polyester resin and 13% for epoxy resin.

The smaller reduction observed for epoxy resin compared to polyester and especially silicone (Figure 6a) suggests that matrices with higher stiffness and cross-link density are more resistant to the presence of filler-induced defects. This indicates a better ability of epoxy resin to maintain structural integrity and partially compensate for stress concentrations caused by the presence of rigid particles.

For elongation-in-break tests, the strength trends were similar. Reference samples (REF) with silicone as a matrix showed an elongation at break of 137.6% (Figure 6b), while filled samples showed a lower elongation at break. The lowest value was recorded for samples containing 7.5% of PCB powder (76.02%). This value was more than 45% lower than the reference sample. The simultaneous decrease in elongation at break (Figure 6b) and tensile strength (Figure 6a) confirms that the addition of WPCB powder leads not only to strength deterioration but also to a significant limitation of deformability. This indicates a transition in the failure mechanism from ductile behavior in reference samples to more brittle fracture in the filled composites, especially silicone-based materials, where polymer chain mobility is strongly restricted by the presence of filler particles.

Similar trends in elongation were observed in break tests for samples with polyester and epoxy resins. A slow reduction in the elongation at break is observed for the samples with increasing amounts of added powder. Again, the smallest value was obtained for samples with the addition of powder at the level of 7.5% and was 0.604 and 0.760% for polyester and epoxy resins, respectively. For comparison, the elongation at break values for the samples without the addition of filler (REF) was 0.850 and 0.933% for the polyester and epoxy resins, respectively (Figure 6b). The decrease in this parameter ranged from about 30% to about 18% for polyester and epoxy resins and was lower than for samples in which silicone was used as a matrix. This behavior further confirms that more rigid and cross-linked matrices, such as epoxy, exhibit lower sensitivity to filler addition, maintaining more stable deformation characteristics compared to polyester and especially silicone (Figure 6b).

In the case of samples with fillers, for all matrixes used, both tensile strength and elongation at break were the highest for samples with the lowest filler content (2.5%). After that, the parameter values slowly decreased. For samples filled with powder in the amount of 2.5 and 5%, the values of tensile strength and elongation at break were similar. Generally, the 7.5% and 10% powder samples had lower tensile strength and elongation at break than the 2.5% and 5% powder filler samples. This trend observed in Figure 6a and Figure 6b suggests the existence of a threshold filler content above which the deterioration of properties becomes more pronounced. It may be associated with increasing particle agglomeration and structural heterogeneity, leading to the formation of additional defects and facilitating crack propagation underload.

For the epoxy resin used as the matrix, much smaller changes in the analyzed parameters were observed than for the polyester resin, when the content of the powder obtained from the recycling of PCBs increased.

#### 3.3.2. Hardness Tests of Composite Materials

The hardness of the tested materials, both for the matrix in the form of silicone and the matrix in the form of resins, increases with an increasing amount of powder added from PCB recycling (Figure 6c). This is especially noticeable in the case of silicone. With 10% powder addition, the hardness increases from 45.4°ShA for the reference sample (REF) to 52.6°ShA. For polyester and epoxy resins, for the addition of powder in the amount of 10% by weight, the hardness increases from 78.2 for the reference sample (REF) to 85.8°ShD and from 82.4 (REF) to 87.8°ShD, respectively. When compared with the results presented in Figure 6a,b, the increase in hardness (Figure 6c) indicates that the WPCB powder acts primarily as a stiffening agent rather than a reinforcing phase. The increase in hardness accompanied by a decrease in strength and elongation suggests that the filler restricts molecular mobility and increases rigidity without improving the load-bearing capacity of the composite. The hardness of the resin-prepared composites was more than two times higher than that prepared on a silicone matrix, which could be expected.

#### 3.3.3. Abrasion Tests of Composite Materials

The results of the tests to evaluate the friction coefficient and abrasion resistance for resins and silicone as a matrix are presented in (Figure 6d). The friction coefficient value for pure polyester resin was 0.324 and the epoxy resin was 0.326. These two values should be treated equally. Similar trends were observed for both polyester resin and epoxy resin. For both resins, the lowest friction coefficient value was recorded for composite materials containing 2.5% filler. Then, as the filler content increased from 5 to 10%, an increase in the friction coefficient was observed. The highest friction coefficient was observed for composites containing 10% filler for epoxy resin and polyester resin. This increase in friction coefficient with filler content (Figure 6d) may be attributed to the presence of hard particles on the contact surface and increased surface roughness, which intensify frictional interactions.

The highest value of abrasive wear was observed for the REF sample (0.82 cm^3^). Material 2.5_F (0.47 cm^3^) has the lowest abrasion resistance value among all tested materials. This value is 43% lower than the REF value. For composite materials with higher filling values, it was observed that with increasing filler content, the abrasion resistance value increases. The abrasion resistance values for materials 7.5_F and 10_F can be treated equally. This behavior indicates that a small addition of filler may initially improve surface stability, while higher filler contents lead to particle pull-out and intensified abrasive wear (Figure 6d).

Considering all the results presented (Figure 6a–d), it can be concluded that the observed changes in mechanical, hardness and tribological properties are governed by common microstructural factors, including the heterogeneous distribution of rigid particles, limited interfacial adhesion, and the formation of stress concentration zones.

### 3.4. Phytotoxicity Tests

**Figure 7:**
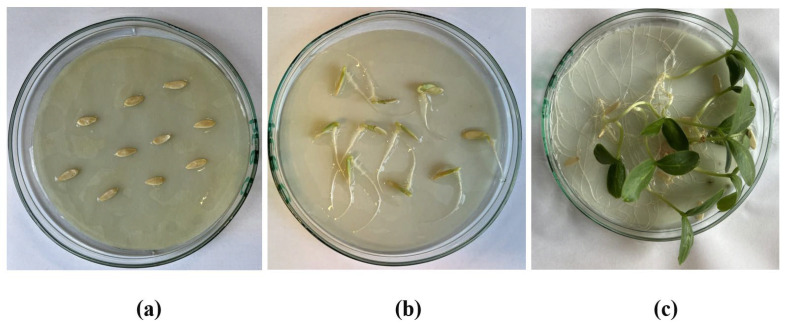
Selected results of phytotoxicity tests. Cucumber in eluate: (**a**) after sowing, (**b**) 5 days after sowing, (**c**) 14 days after sowing.

Figure 7 shows the results of example phytotoxicity tests. From the phytotoxicity tests, it was found that all plants germinated 5 days after sowing. Importantly, after 14 days, no inhibition of root or stem growth was observed. In contrast, in most cases, an increase in root and stem length was observed in plants watered with eluate compared to plants watered with distilled water, which does not contain nutrients that may be available in the eluates. This is what happens if they do not contain toxic substances. In general, the increase in root length was greater than the increase in stem length of the studied plants and amounted to 163%, 218%, and 192% for cucumber, mustard, and wheat, respectively. The increase in stem length for these plants was 131%, 139%, and 94%, respectively. Only in the case of the length of the wheat stem was a growth inhibition of 6% observed. Therefore, it can be concluded that the tested plants were not sensitive to substances leached from plastic. In contrast, the substances presented in the eluates promoted the growth of the tested plants.

## 4. Economic, Material, Technical, and Environmental Analysis

During physical recycling, three products are created: concentrate, a mixture of metals, waste, plastics with a small amount of fiberglass, and an intermediate-middling’s product, i.e., particles containing a mixture of plastics and metals. The concentrate obtained, due to its high purity and valuable element content, can be sold to metal producers or processors and represents a significant financial benefit to the WPCB recycler. Given the current metal prices and the average metal content of WPCB, it is estimated that in one ton of WPCB, the price of the metals contained is more than USD 91,800. This is based on metal prices from 2025 [8,10]. This is, of course, the value of metals for unprocessed WPCBs. It should be noted that, despite the small quantities, gold is the key metal determining the economic viability of WPCB recycling. However, since the metal content of WPCB varies depending on the producer, the year of production, and the technology used, recycling of other metals should not be ruled out as a predictable source of financial benefit.

In Poland, the annual weight of the collected WPCB per 1 million inhabitants is estimated to be about 27 Mg (mass without sockets, resistors, transistors, processors, RAM chips, and other elements on the surface of the PCB), with an estimated value of metals of about USD 2.5 million/year (the mass balance of WPCB and the estimated value of metals and plastics in WPCB were determined on the basis of data from three companies located in the Silesian Voivodeship dealing with WEEE collection). For example, in the Silesian Voivodeship, where 4.4 million people live, potentially about 120 Mg of WPCB are collected annually, and the value of the metals can be estimated at USD 11 million. Therefore, this represents a major opportunity for local WEEE recyclers. For the whole of Poland, the value of metals is even USD 96 million. Because of the high purity of the generated products, the valuable metal mixture can be sold to local metal smelters, while the product consisting of the nonmetallic parts can be used as a filler to produce composite materials based on polymer materials with the addition of fillers from powder derived from WPCB recycling. The method of manufacturing composites has already been submitted to the Patent Office of the Republic of Poland [23]. Because the addition of recycled plastics improved abrasion resistance and hardness in each sample, especially epoxy resins as a matrix (Figure 6c,d), these materials could be used in the production of resin floors and composite boards. This improvement is associated with the stiffening effect of rigid WPCB particles, which increase surface resistance and limit material wear. Composite materials with the addition of recycled plastics have slightly lower tensile strength for some materials depending on the matrix type (by only about 15% for polyester resin, 13% for epoxy resin and 48% for silicone). The reduction, observed in (Figure 6a), might be related to limited interfacial adhesion between the matrix and the filler, leading to stress concentration and ineffective load transfer. A similar decreasing trend in elongation break (Figure 6b) indicates that the addition of WPCB powder also reduces deformability, causing a transition toward more brittle behavior, particularly in the case of silicone. The results also suggest that low filler content (2.5–5%) provides a compromise between maintaining mechanical performance and improving hardness and tribological properties, while higher filler content leads to increased structural heterogeneity and further deterioration of strength-related parameters. Therefore, these should not be used in products that are exposed to significant mechanical loads or dynamic stress. However, they may be suitable for applications where stiffness, hardness and wear resistance are more critical than strength. More importantly, consumer products manufactured in this way are not toxic to plants, which additionally supports their potential use as environmentally friendly materials incorporating recycled waste. The addition of up to 10% powder to epoxy resin to industrial floor screeds is recommended, as it enhances hardness and wear resistance. The annual mass of recycled plastics for the Silesian Voivodeship in Poland reaches almost 20 tons per million inhabitants per year. This is a significant amount and could mean that fewer raw materials will need to be purchased to produce new consumer goods such as resin flooring and composite decking.

**Figure 8:**
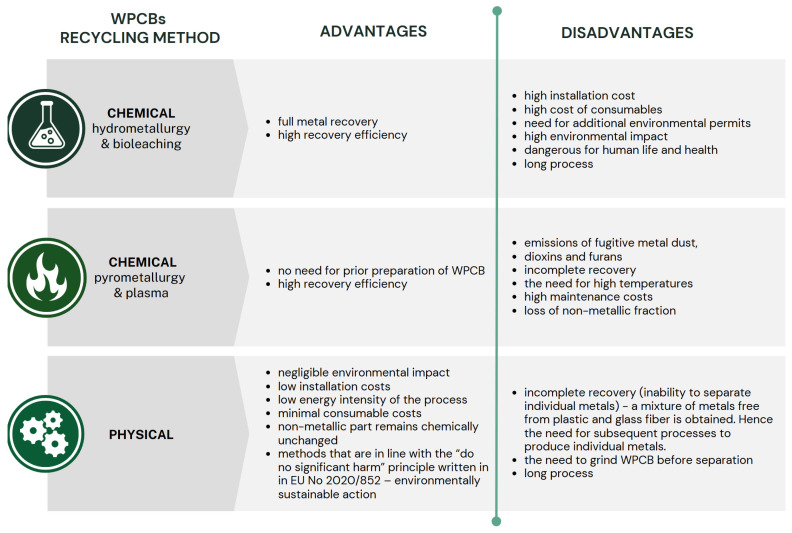
The main advantages and disadvantages of WPCB recycling methods, based on [6,19,45,46,47].

In summary, recycling using physical separation methods is relatively easy to perform and does not require sophisticated technical equipment or great financial expenditures. It also has a small impact on the environment as discussed before (Figure 8). One of the most important processes preceding the separation methods is comminution, whose purpose is to liberate metals from the rest of the WPCB substrates. Improper comminution of the WPCB results in a certain randomness in the physical properties of the particles, which is the main reason for inefficient separation and thus the migration of the particles into the incorrect products. Therefore, release of metals from the remaining non-metallic fractions is crucial to achieve high selectivity and efficiency in separation. WPCB grinding usually involves several steps which include shredding, hammer mills or knife mills as studied here. The resulting WPCB powder is the feed for the separation processes. Separation in the electrostatic separator occurs as a result of differences in the electrical surface charge of various particles and separation is carried out without the use of process water. Because of its high efficiency in separation and selectivity and the smallest environmental footprint, electrostatic separation is the best option for separating metals from plastics.

**Figure 9:**
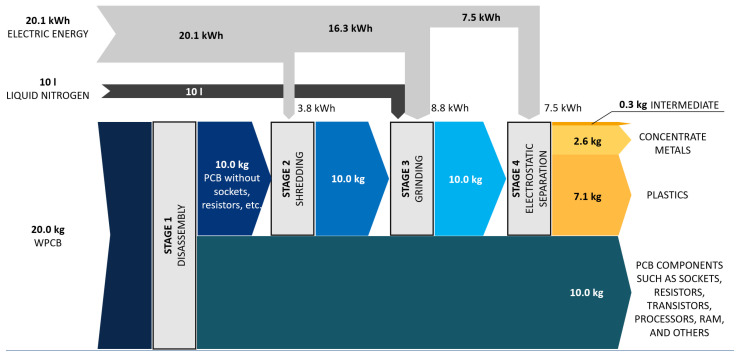
Technology diagram with mass and energy flows presented using a Sankey diagram.

Currently, in many countries, WPCB recycling is carried out exclusively by chemical methods, i.e., hydrometallurgy, pyrometallurgy, and/or biohydrometallurgy. Their use generally requires special permits and additional processing steps to prevent great environmental impact. It should also be noted that the chemical transformations during WPCB recycling processes result in heavy cross-contamination of non-metallic parts, which in turn creates additional waste stream (Figure 8). In contrast to the above, methods based on physical separation have less impact on the natural environment [19,24]. These processes do not use hazardous substances that cause significant emissions into the atmosphere as a result the carbon footprint for physical recycling is relatively small. By using physical separation methods for recycling allows the recovery of valuable substances (metals) and the use of the non-metal fraction (fiberglass and plastics) without cross-contamination for the manufacturing of new products. Thus, physical methods provide opportunities for close to zero-waste recycling of WPCB. In addition, the non-metallic part of the ground WPCB laminate is not chemically altered, creating a wide range of application possibilities, for example, for the production of composite materials. Figure 9 shows a flow diagram of the eco- efficient WPCB recycling technology with mass and energy flows presented using the Sankey diagram. However, it should be noted that the use of physical methods does not allow for full metals recovery, i.e., extraction of metals in the pure metallic form. Since a mixture of metals is created from which individual metals can be further obtained by the follow up chemical treatment. Due to the well-developed metallurgical industry in Poland, it is possible to transfer (sell) such metal mixture from PCB recovery to existing metal production or recovery plants.

During such processing, there is about 2.4% of the intermediate product of middling’s quality and because of such a small quantity of this product, it is possible to process it by bioleaching, as indicated in the Section 3.1. Although bioleaching is characterized by slow kinetics and is generally a low-yielding method, it is still more environmentally friendly method than chemical leaching (hydrometallurgy). Alternatively, this material can be sent directly to chemical leaching, and because of its low mass, it should have a relatively smaller negative impact on the environment because less chemicals will be used for smaller quantity of this fraction.

In order for a company to be able to recover metals in accordance with the presented method, it is necessary to additionally consider investment, labor, and administrative costs, including taxes, as well as process costs related to supplying machines and devices with electricity and the use of liquid nitrogen, as well as the management of other products generated during recycling. Table 1 shows the input used in the LCA of each stage, along with the unit prices of the input, and the calculated consumable costs required to process 1 kg of WPCB. The calculations were made for devices operating on a laboratory scale; hence, the consumable costs are high. Without these costs, the most expensive stage is cryogenic grinding, mainly due to the price of liquid nitrogen.

## 5. Conclusions

Physical WPCB recycling technology using shredding, cryogenic grinding, and electrostatic separation is in line with the principles of circular economy and sustainable production. It is a pretreatment method that complements conventional chemical recycling methods.

The global warming potential for the entire physical and chemical process is lower by about 70%, which is due to the smaller mass of input material going to the downstream metallurgical processes. Because of this, the entire recycling process generates less waste and uses fewer leaching agents (for hydrometallurgy). In addition, plastics produced through physical recycling can be transform into powders and used to produce consumer goods and thus even bring financial benefits, similar to selling the metal mixture itself. By addition of PCB powder to resins and silicone it can improve mechanical properties.

Future development of PCB recycling will focus on advanced, environmentally friendly technologies that improve the recovery of valuable materials and reduce hazardous waste. Increasing automation, stricter environmental regulations, and a circular economy will also play a key role in making PCB recycling more efficient and sustainable. Additionally, the use of waste to create composite materials and conducting research on them contributes to significant development of materials engineering in the context of expanding the possibilities of creating increasingly better materials for the future.

## Figures and Tables

**Table 1:** Economic analysis of processing 1 kg of WPCB including operation and maintenance costs

Input	Unit Price, USD	Consumption/Price, USD		
		Shredding	Cryogenic Grinding	Electr. Separat.
Electricity [48], kWh	0.012	0.005	0.011	0.009
Liquid nitrogen, liters	0.59		0.59	
Consumable costs		0.49 ^1^	1.02 ^2^	<0.001 ^3^
Price excl. consumable costs, USD		0.005	0.601	0.009
Total price, USD		0.495	1.621	0.009

^1^ Cutting shaft elements (36 pcs/2 Mg_wpcb_); ^2^ mill knives (7 pcs/100 kg_wpcb_); ^3^ electrode (1 pc/5 Mg_WPCB_).

## Data Availability

The original contributions presented in this study are included in the article. Further inquiries can be directed to the corresponding author.

## Appendix A

**Table A1:** Life cycle inventory to process 1 kg WPCB

Stages			Energy Consumption, kWh/kg WPCB	Water Consumption, dm^3^/kg WPCB	Liquid Nitrogen Consumption, dm^3^/kg WPCB	Flotation Reagent Consumption, mg/kg WPCB
Shredding			0.375			
Grinding		Cooling			1	
		Grinding	0.880			
Separation	Electrostatic Separator		0.750			
	Shaking Table	Separation	0.333	150		
		Drying	1.200			
	Cyclofluid Separator	Separation	0.093	50		
		Drying	1.200			
	Flotation Machine	Separation	0.552	30		3140
		Drying	1.200			
